# Investigation of a new implant surface modification using phosphorylated pullulan

**DOI:** 10.3389/fbioe.2024.1378039

**Published:** 2024-05-22

**Authors:** Kanako Nagamoto, Ko Nakanishi, Tsukasa Akasaka, Shigeaki Abe, Kumiko Yoshihara, Mariko Nakamura, Hiroshi Hayashi, Shinji Takemoto, Masato Tamura, Yoshimasa Kitagawa, Bart Van Meerbeek, Yasuhiro Yoshida

**Affiliations:** ^1^ Oral Diagnosis and Medicine, Faculty of Dental Medicine, Hokkaido University, Sapporo, Japan; ^2^ Department of Biomaterials and Bioengineering, Faculty of Dental Medicine, Hokkaido University, Sapporo, Japan; ^3^ BIOMAT, Department of Oral Sciences, KU Leuven, Leuven, Belgium; ^4^ Department of Dental and Biomedical Materials Science, Graduate School of Biomedical Sciences, Nagasaki University, Nagasaki, Japan; ^5^ Health and Medical Research Institute, National Institute of Advanced Industrial Science and Technology, Takamatsu, Japan; ^6^ School of Clinical Psychology, Kyushu University of Medical Science, Miyazaki, Japan; ^7^ Section for Dental Innovation, Faculty of Dental Medicine, Hokkaido University, Sapporo, Japan; ^8^ Department of Biomedical Engineering, Iwate Medical University, Shiwa, Japan; ^9^ Department of Oral Biochemistry and Molecular Biology, Graduate School of Dental Medicine, Hokkaido University, Sapporo, Japan

**Keywords:** phosphorylated pullulan, dental implants, surface modification, cellular response, titanium

## Abstract

Various implant surface treatment methods have been developed to achieve good osseointegration in implant treatment. However, some cases remain impossible to treat with implants because osseointegration is not obtained after implantation, and the implants fail. Thus, this study focused on phosphorylated pullulan because of its adhesiveness to titanium (Ti) and bone, high biocompatibility, and early replacement with bone. In this study, the response of bone-related cells to phosphorylated pullulan was evaluated to develop a new surface treatment method. Saos-2 (human osteosarcoma-derived osteoblast-like cells), MC3T3-E1 (mouse calvaria-derived osteoblast-like cells), and RAW264.7 (mouse macrophage-like cells) were used. In evaluating cellular responses, phosphorylated pullulan was added to the culture medium, and cell proliferation and calcification induction tests were performed. The proliferation and calcification of cells on the surface of Ti disks coated with phosphorylated pullulan were also evaluated. In addition, bone morphogenetic protein-2 (BMP-2), an osteogenic factor, was used to evaluate the role of phosphorylated pullulan as a drug carrier in inducing calcification on Ti disks. Phosphorylated pullulan tended to promote the proliferation of osteoblast-like cells and the formation of calcification on Ti disks coated with phosphorylated pullulan. Ti disks coated with phosphorylated pullulan loaded with BMP-2 enhanced calcification. Phosphorylated pullulan inhibited osteoclast-like cell formation. These results are due to the properties of phosphorylated pullulan, such as adhesiveness to titanium and drug-loading function. Therefore, phosphorylated pullulan effectively promotes bone regeneration when coated on titanium implants and is useful for developing a new surface treatment method.

## 1 Introduction

Since the report of Brånemark regarding osseointegration between bone and titanium (Ti) (Block, 2018), various improvements have been made to achieve good osseointegration (Bosshardt et al., 2017). Surface treatment of implants is one of them. Surface treatments include anodizing, acid etching, sandblasting, TiOblasting (Collaert et al., 2008), titanium plasma-spraying (Becker et al., 2000), hydroxyapatite coating (Smeets et al., 2016), and others. These treatment processes have been clinically applied as products. However, there are still many cases wherein sufficient osseointegration cannot be obtained, and implants drop out prematurely. In addition, hydroxyapatite coating results in loosening of the coating, which causes peri-implantitis, and bone resorption that occur after implantation (Albrektsson, 1998). Furthermore, risk factors in implant treatment include diabetes, radiation therapy, smoking, periodontal disease, and bruxism, which can make treatment more difficult (Albrektsson, 1998; Kullar and Miller, 2019). Therefore, improvement of implant surface properties, which achieve sufficient osseointegration acquisition, an affinity for titanium and bone, high biocompatibility, and early bone replacement properties, requires new coating materials (Choi et al., 2013; Abraham, 2014; Smeets et al., 2016; Chouirfa et al., 2019; Wegener et al., 2020).

Pullulan is polysaccharide consisting of α-(1,6) linked maltotriose composed of three molecules of glucose bonded α-(1,4). It is produced from sucrose or starch by *Aureobasidium pullulans*. The substance has excellent biosafety (Aydogdu et al., 2016) and adhesive properties and is used in various fields, such as food and pharmaceuticals. Furthermore, phosphorylated pullulan has excellent biocompatibility, and it can promote bone regeneration and serve as a drug carrier (Yonehiro et al., 2013). Phosphorylated pullulan also promoted good osseointegration when implants were coated with phosphorylated pullulan and implanted in the parietal bone of a miniature pig (Cardoso et al., 2017). Bone formation was similarly enhanced when phosphorylated pullulan-coated Ti disks were implanted in rabbit tibia (Cardoso et al., 2014). However, few reports have been found on the effects of phosphorylated pullulan on bone-related cells in cell experiments.

In this study, phosphorylated pullulan was added to the culture medium or adsorbed to Ti disks, and then bone-related cells were cultured by using them for evaluation of cell proliferation and differentiation induction. Differentiation induction was also evaluated to investigate the drug carrier ability of phosphorylated pullulan when phosphorylated pullulan was loaded with a bone formation factor.

## 2 Materials and methods

### 2.1 Preparation of the phosphorylated pullulan

**FIGURE 1:**
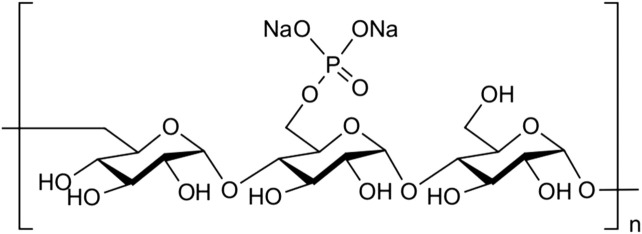
Structure of phosphorylated pullulan. Phosphate groups were attached to the carbon at position six of the glycosidic-bonded glucan at α1-4 and α1-6 to form pullulan.

Pullulan (average molecular weight of 322,916; Hayashibara, Inc., Okayama, Japan) was dissolved in sodium hydroxide aqueous solution and stirred at 20°C overnight; after cooling to 0°C, phosphoryl chloride was added, and the solution was stirred at 0°C for 6 h. The solution was dialyzed to remove sodium phosphate and sodium chloride by-products. After concentration, the solution was lyophilized to produce phosphorylated pullulan (average molecular weight of 617,515; Figure 1).

### 2.2 Phosphorylated pullulan treatment on Ti disks (coating of phosphorylated pullulan on Ti disks)

#### 2.2.1 Observation of Ti disks

Ti disks (JIS class 2 pure titanium, GC Corporation, Tokyo, Japan) were randomly ground with a #400 waterproof abrasive paper in 5-cm squares for 4 min. The same method was used for the following grinding. The ground Ti disks were observed using a scanning electron microscope (SEM, S4800, Hitachi High-Tech Corporation, Tokyo, Japan) and a shape-measuring laser microscope (VK-X200, Keyence Corporation, Osaka, Japan).

#### 2.2.2 Confirmation of the phosphorylated pullulan adhesion on Ti disks

The Ti disks were ground using the same method. The ground Ti disks were immersed in 99.5% acetone for 1 h and then in 99% ethanol for 30 min. Immersion was repeated three times. The obtained Ti disks were immersed in 1% phosphorylated pullulan solution using MilliQ as a solvent for 24 h. Then, the disks were ultrasonically washed in deionized water for 10 min, and this process was repeated three times. The controls were immersed in MilliQ for 24 h and washed in the same manner.

In confirming the adhesion of phosphorylated pullulan to Ti disks, the disks were analyzed by X-ray photoelectron spectroscopy (XPS, Axis-Ultra, Shimadzu Corporation, Kyoto, Japan).

#### 2.2.3 Observation of cells on Ti disks coated with phosphorylated pullulan

The Ti disks were soaked in 99.5% acetone, 99% ethanol, and 0.5% hydrochloric acid and sonicated for 10 min. Afterward, the disks were sonicated two times with deionized water for 10 min. The disks were sterilized in an autoclave. After immersion in 1% phosphorylated pullulan solution using MilliQ as a solvent for 24 h, the disks were washed three times with deionized water. The control disks were immersed in MilliQ instead of 1% phosphorylated pullulan solution for 24 h and washed in the same manner. Ti disks were placed on 24-well plates and seeded with Saos-2 at a concentration of 7.5 × 10^4^ cells/well. The medium was Dulbecco’s Modified Eagle Medium (DMEM) with high glucose (Sigma-Aldrich Co. LLC., St. Louis, United States of America) containing 10% FBS and 1% penicillin–streptomycin amphotericin B. The cells were incubated for 30 min at 37°C under 5% CO_2_.

After 30 min, the disks were washed and immersed in 1% glutaraldehyde to fix the cells. The cells were then dehydrated using an alcohol series (50%, 60%, 70%, 80%, 90%, 95%, 99.5%, and 100%), and critical point drying was performed. The samples were pretreated with Pt–Pd conductors using an ion sputtering system (E1030, Hitachi High-Tech Corporation, Tokyo, Japan). The samples were observed using a SEM at a pressurized voltage of 10 kV.

### 2.3 Cell proliferation test

#### 2.3.1 Cell culture using a culture medium, including phosphorylated pullulan

MEM-α (FUJIFILM Wako Pure Chemical Corporation, Osaka, Japan) containing 10% FBS and 1% penicillin–streptomycin–amphotericin B was prepared as the culture medium. MC3T3-E1 cells were seeded at a concentration of 1 × 10^3^ cells/well in 96-well plates and cultured for 1 day at 37°C under 5% CO_2_. The culture medium was aspirated and then replaced with a new medium, including phosphorylated pullulan at concentrations of 0% (control), 0.01%, 0.1%, 0.5%, 1%, and 3%, and incubated for 2 days. Using the Cell Counting Kit-8 (WST-8, Dojindo Laboratories, Kumamoto, Japan), the number of cells was evaluated indirectly. Absorbance was measured using a microscope reader (Spectra Max Paradigm, Thermo Fisher Scientific, Waltham, United States of America) at 450 nm. Dunnett’s *t*-test was used for statistical processing between control and other conditions, and the statistically significant difference was set to *p* < 0.05.

#### 2.3.2 Cell culture using Ti disks coated with phosphorylated pullulan

The ground Ti disks were immersed in 99.5% acetone for 1 h and then in 99% ethanol for 30 min. These processes were repeated three times. Then, the disks were ultrasonically cleaned three times in deionized water for 10 min and immersed in 1% phosphorylated pullulan solution using MilliQ as a solvent for 24 h. The disks were sonicated in deionized water for 10 min and repeated three times. The control disks were immersed in MilliQ for 24 h and washed in the same manner. The disks were then sterilized in an autoclave. Phosphorylated pullulan-coated Ti disks and control Ti disks were placed on 24-well plates and seeded with MC3T3-E1 at a concentration of 1 × 10^4^ cells/well. The cells were cultured for 1 or 2 days. The culture medium was MEM-α containing 10% FBS and 1% penicillin–streptomycin–amphotericin B. The cells were cultured at 37°C under 5% CO_2_. The Ti disks were washed once with PBS, avoiding the stream from hitting the Ti disks directly. The Ti disks were transferred to another plate filled with a fresh medium, and 50 µL of WST8 solution was added to each well. The cells were then incubated. The number of cells was evaluated by measuring the absorbance at 450 nm using a microplate reader. Student’s t-test was used for statistical processing between the two conditions, and the statistically significant difference was set to *p* < 0.05.

### 2.4 Effect of phosphorylated pullulan on the induction of calcification

#### 2.4.1 Cell culture using a culture medium, including phosphorylated pullulan

Saos-2 was seeded into 24-well plates at a concentration of 2.3 × 10^4^ cells/well and cultured for 5 days under the same conditions as described in Section 2.2.3. Culturing was continued for another week under three conditions using three different media, namely, calcification induction medium, calcification induction medium mixed with phosphorylated pullulan at a concentration of 1%, and DMEM medium as a control. The calcification induction medium was DMEM with supplements. The final concentrations of the supplements were 10 nM dexamethasone, 50 μg/mL ascorbic acid, and 5 mM β-glycerophosphate. The culture was performed at 37°C under 5% CO_2_ in the air phase, and the medium was changed after 3 or 4 days. After incubation, each well was washed with PBS. Cells were fixed with methanol and washed again. Then, the cells were stained with an alizarin red solution and washed several times with deionized water.

#### 2.4.2 Cell culture using Ti disks coated with phosphorylated pullulan

The ground Ti disks were soaked in 99.5% acetone, 99% ethanol, and 0.5% hydrochloric acid and sonicated for 10 min. Afterward, the disks were sonicated two times with deionized water for 10 min. The disks were sterilized in an autoclave. After immersion in 1% phosphorylated pullulan solution using MilliQ as a solvent for 24 h. The control Ti disks were immersed in MilliQ for 24 h instead of 1% phosphorylated pullulan. Phosphorylated pullulan-coated Ti disks and control Ti disks were placed on 24-well plates and seeded with Saos-2 at 6.48 × 10^4^ cells/well for 3 days under the same conditions described in Section 2.2.3. The Ti disks were transferred to new 24-well plates filled with a calcification induction medium and incubated for 2 weeks. The Ti disks were washed; the cells were fixed, and calcification formation was stained using the same methods as described in Section 2.4.1.

### 2.5 BMP-2 loading effect using phosphorylated pullulan on Ti disks

The ground Ti disks were washed and sterilized using the same process described in Section 2.4.2. The Ti disks were immersed in 0.2 μg/mL BMP-2 (Proteintech Group, Inc., Rosemont, United States of America) + 1% phosphorylated pullulan solution or 0.2 μg/mL BMP-2 solution for 1 day. The disks were washed three times, transferred to a new plate, seeded with Saos-2 at a concentration of 1 × 10^5^ cells/well, and incubated for 3 days under the same conditions as described in Section 2.2.3. The disks were transferred to a new plate and replaced with a calcification induction medium, and the concentration of β-glycerophosphate was adjusted to 10 mM. The concentrations of the other supplements such as dexamethasone and ascorbic acid were not changed from Section 2.4. The cells were cultured for 4 days for calcification induction in the same way as described in Section 2.4.1. The cells were washed, fixed, and stained using the same methods as described in Section 2.4.1.

### 2.6 Effect of phosphorylated pullulan on osteoclast-like cell formation

RAW264.7 were seeded at a density of 5,000 cells/cm^2^ in 24-well plates and grown for several days. MEM-α containing 10% FBS supplemented with 1% penicillin–streptomycin–amphotericin B was used as the medium. The cells were cultured at 37°C under 5% CO_2_ atmosphere. Next, 50 ng/mL of receptor activator of nuclear factor-kappa B ligand (RANKL, sRANKL, Oriental Yeast, Tokyo, Japan) and phosphorylated pullulan were added at 0% (control), 0.1%, 1%, and 3%, and the cells were incubated at 37°C for 6 days under 5% CO_2_ atmosphere. The medium was changed every 2 days. After culture, cells were washed with PBS, fixed with 4% paraformaldehyde (FUJIFILM Wako Pure Chemical Corporation, Osaka, Japan), washed with distilled water, and stained for osteoclast-like cells using the TRAP staining kit (Cosmo Bio Company, Inc., Tokyo, Japan). Osteoclast-like cells were defined as TRAP-positive cells with three or more nuclei and were observed and measured. Osteoclast-like cells were randomly counted in 20 fields of view. Dunnett’s *t*-test was used for statistical processing between control and other conditions, and the statistically significant difference was set to *p* < 0.05.

## 3 Result

### 3.1 Phosphorylated pullulan treatment of Ti disks

#### 3.1.1 SEM

**FIGURE 2:**
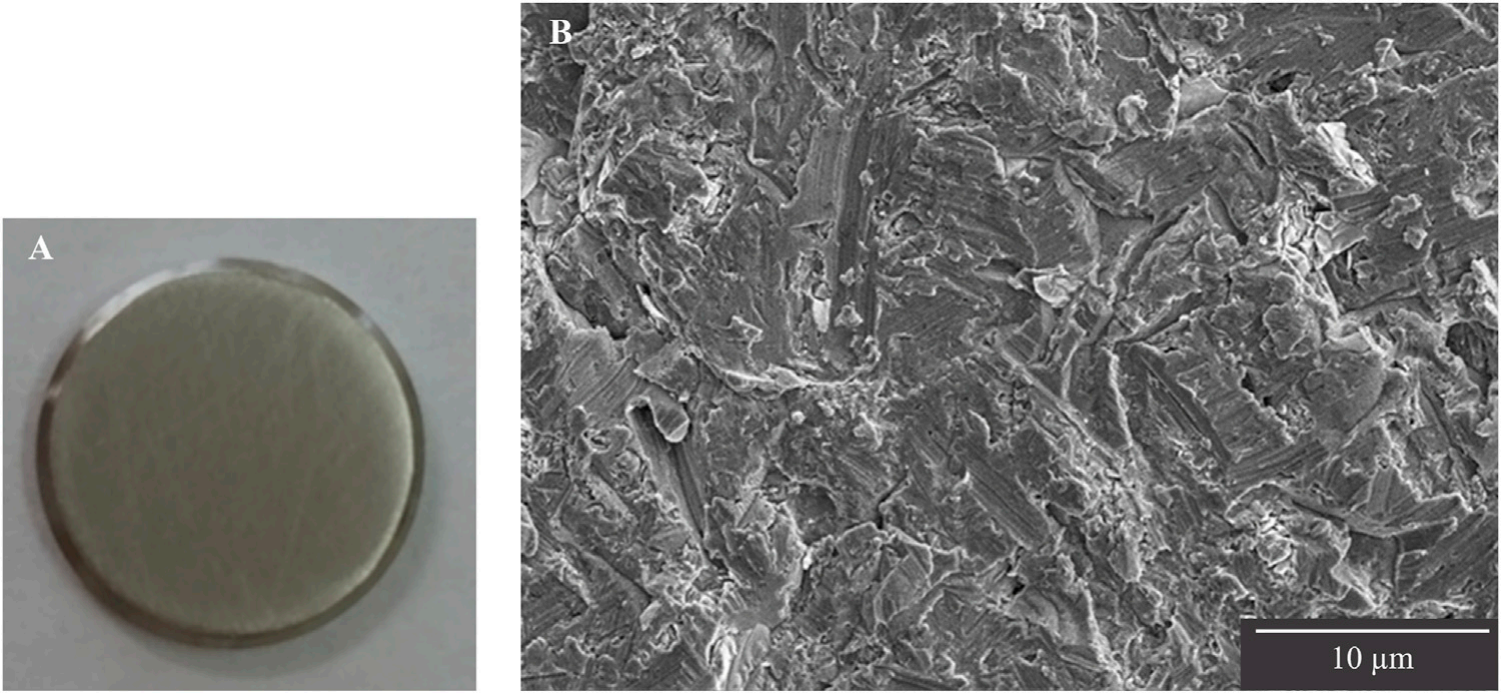
Observation of Ti disk surfaces after grinding. **(A)** Image of Ti disk ground with #400 waterproof abrasive paper. **(B)** SEM image of Ti disk surface ground up to #400.

The surfaces of Ti disks ground in random directions with #400 waterproof abrasive paper of 5-cm square were observed by SEM, and the entire surface was uniformly ground without any large scratches (Figure 2).

#### 3.1.2 Shape measurement laser microscope

**FIGURE 3:**
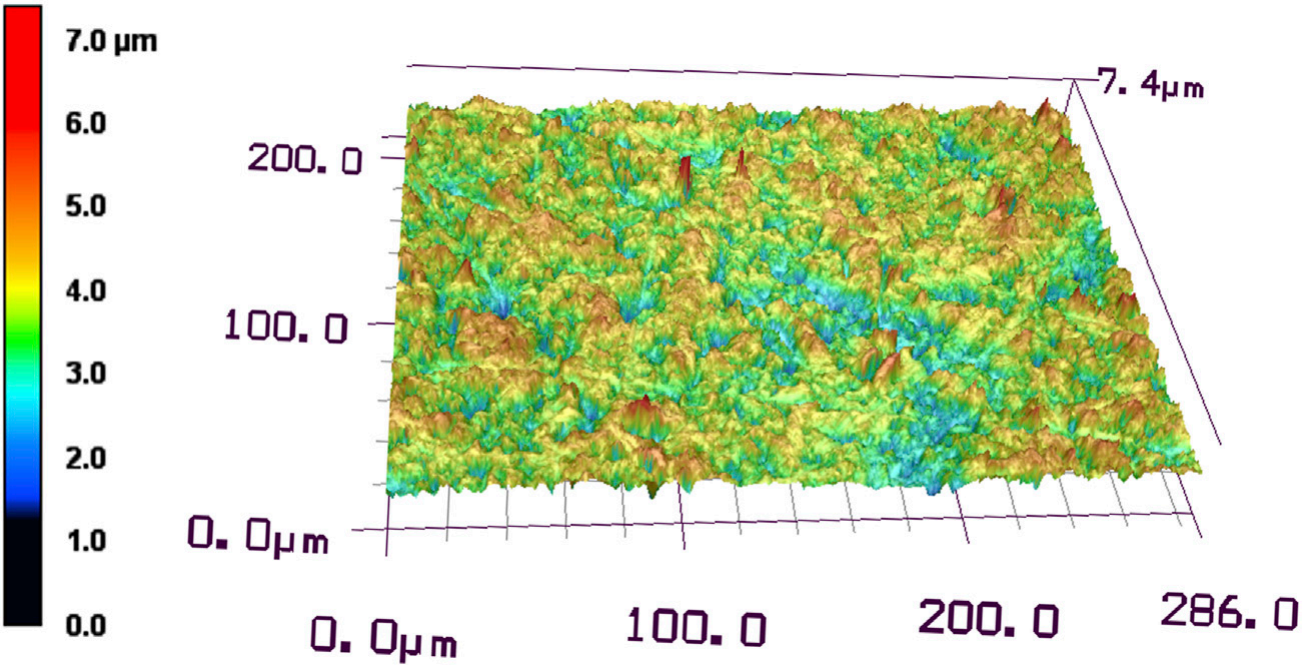
Laser microscopic observation of Ti disk surface after grinding with #400 waterproof paper.

The surface roughness of the disk ground with #400 waterproof paper was examined using a laser microscope. The Ra of the disk ground with #400 waterproof paper was 0.47 ± 0.04 µm. The 3D images showed that the top surface of the disk ground with #400 waterproof paper had a height difference of 7.4 µm (Figure 3).

#### 3.1.3 XPS

**FIGURE 4:**
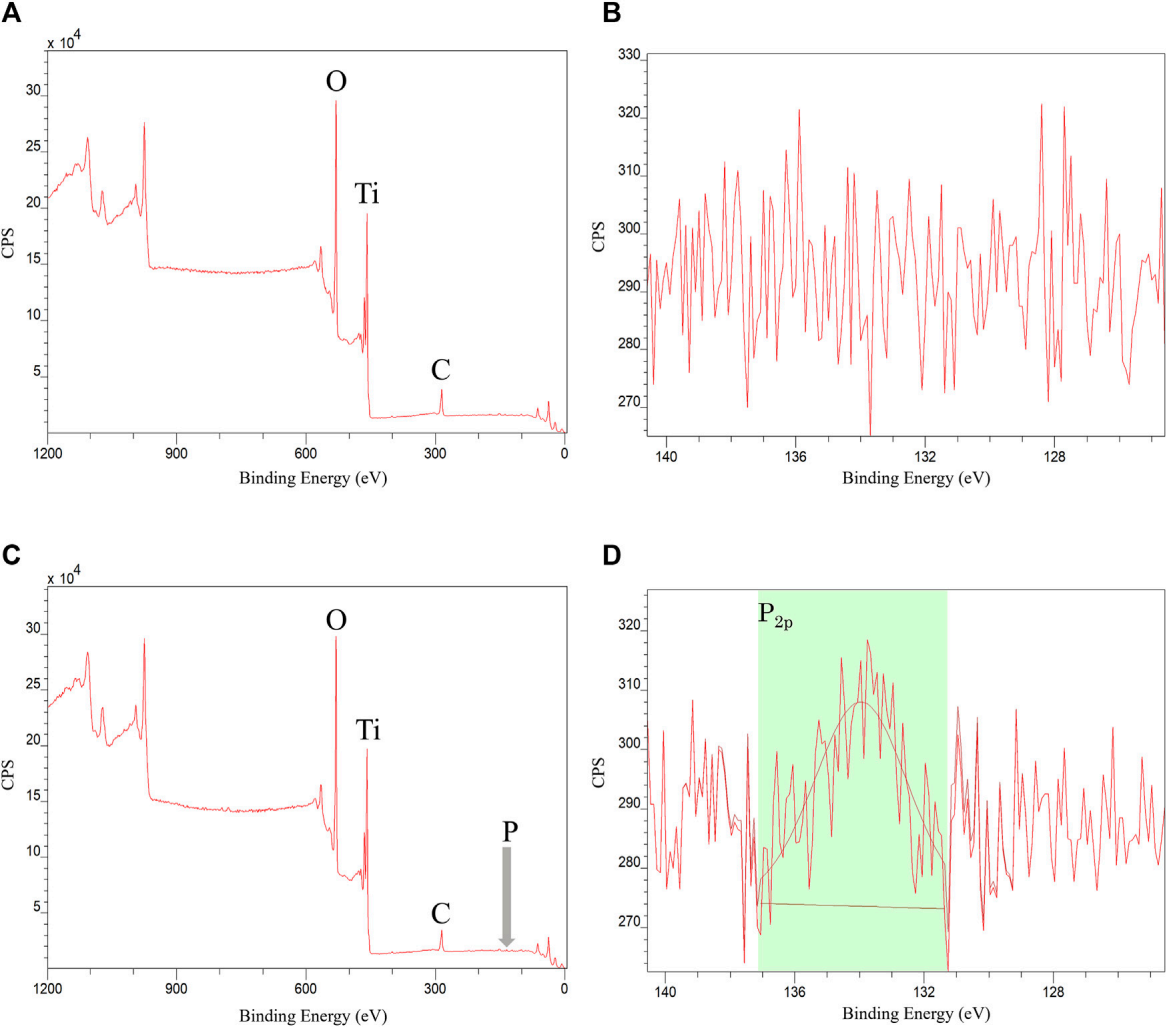
XPS measurement of Ti disk. **(A,B)** Control Ti disk. **(C,D)** Phosphorylated pullulan-coated Ti disk.

XPS analysis of the Ti disk surface treated with phosphorylated pullulan showed a peak at approximately 134 eV. This signal was derived from phosphorus (P2p), and the presence of phosphate groups derived from phosphorylated pullulan on the Ti disk surface was confirmed. The composition of phosphorus on the Ti disk surface treated with phosphorylated pullulan was 0.5–0.9 atomic% of the total percentage of elements detected. This result indicates that the phosphorylated pullulan adsorbed to the Ti disk surface (Figure 4).

### 3.2 Observation of cells on the phosphorylated pullulan-treated Ti disk surface

**FIGURE 5:**
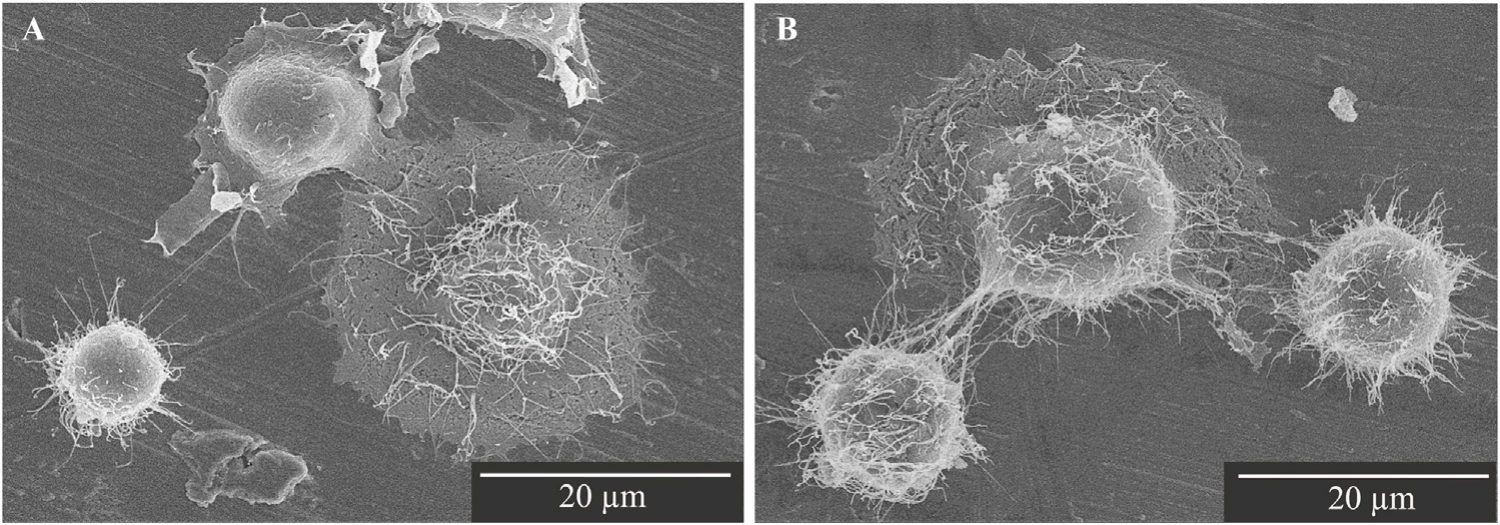
SEM image of Saos-2 on the Ti disks at ×2,000 magnification. Scale bar = 20 μm. **(A)** Control Ti disk. **(B)** Phosphorylated pullulan-coated Ti disk.

Saos-2 cells cultured on the Ti disks for 30 min were observed to adhere to the surface of the Ti disks by SEM. Lamellipodia and filopodia were observed on the cell on each Ti disks, which are involved in cell body migration and probing the surrounding environment (Mattila and Lappalainen, 2008). Cells cultured on phosphorylated pullulan-treated Ti disks seem to have more filopodia than control Ti disks (Figure 5).

### 3.3 Cell proliferation test

#### 3.3.1 Cell culture using a culture medium, including phosphorylated pullulan

**FIGURE 6:**
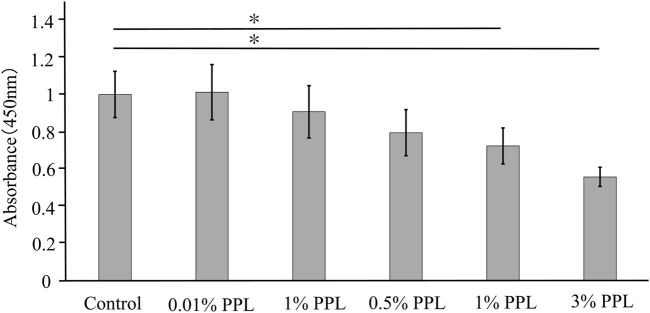
Cell proliferation test using medium supplemented with phosphorylated pullulan. The cells were MC3T3-E1. Control in the graph showed the medium without phosphorylated pullulan. PPL in the graph showed the medium supplemented with phosphorylated pullulan. Significance difference test: *p < 0.05.

The direct addition of phosphorylated pullulan to the culture medium at concentrations of 1% and 3% significantly inhibited the cell proliferation of MC3T3-E1 compared with the control (Figure 6).

#### 3.3.2 Cell culture using phosphorylated pullulan-coated Ti disks

**FIGURE 7:**
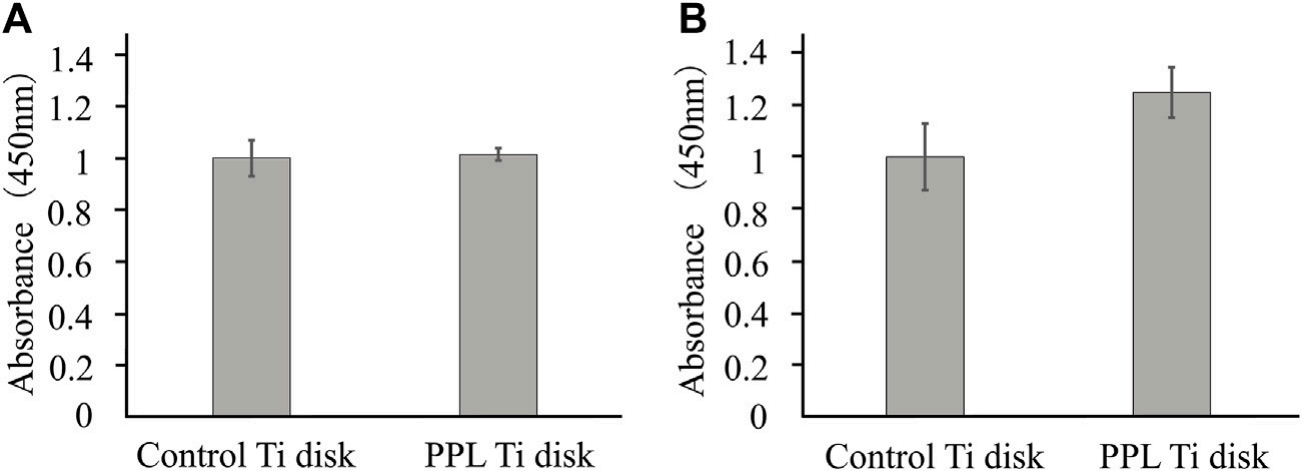
Cell proliferation test on Ti disk surface. The cells were MC3T3-E1. **(A)** Day 1. **(B)** Day 2.

On day 1, the number of MC3T3-E1 cells did not change, but on day 2, the number of MC3T3-E1 cells tended to increase on the phosphorylated pullulan-coated Ti disks compared with the uncoated Ti disks, although the difference was not statistically significant (Figure 7).

### 3.4 Effect of osteoblast-like cells on the induction of calcification

#### 3.4.1 Cell culture using a culture medium, including phosphorylated pullulan

**FIGURE 8:**
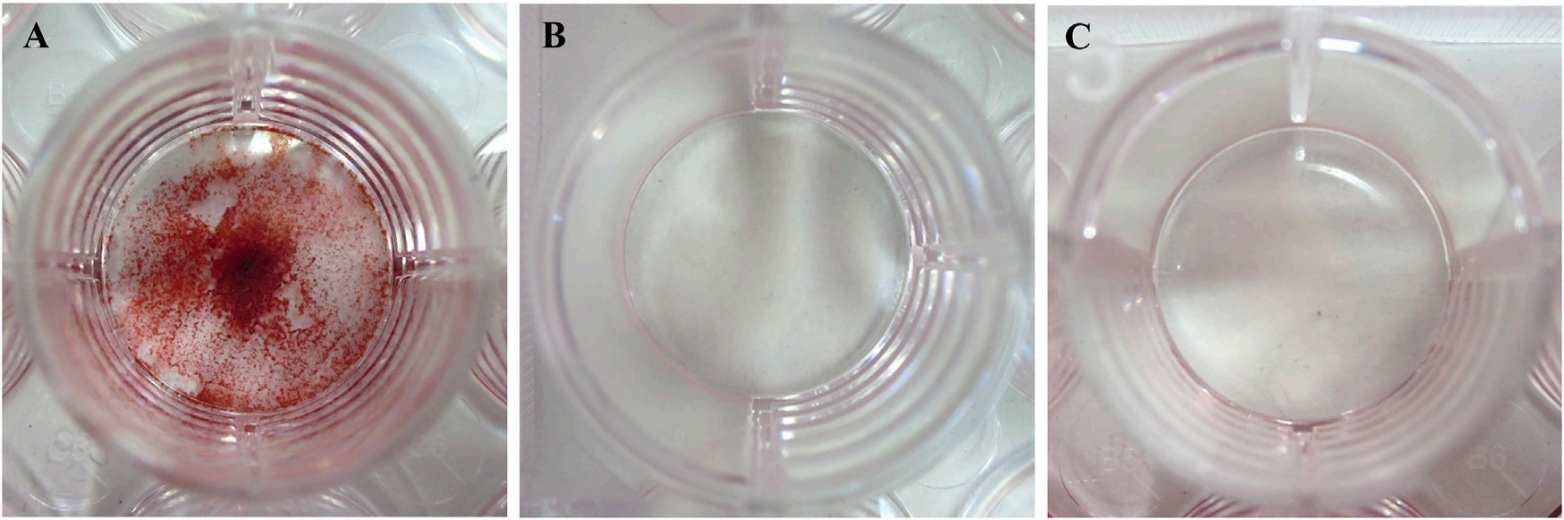
Induction of calcification. The cells were Saos-2. **(A)** Calcification induction medium. **(B)** Calcification induction medium supplemented with 1% phosphorylated pullulan. **(C)** DMEM medium.

Saos-2 produced calcification products in the calcification induction medium group, whereas no calcification occurred in the DMEM and calcification-inducing medium groups containing 1% phosphorylated pullulan (Figure 8).

#### 3.4.2 Cell culture using phosphorylated pullulan-coated Ti disks

**FIGURE 9:**
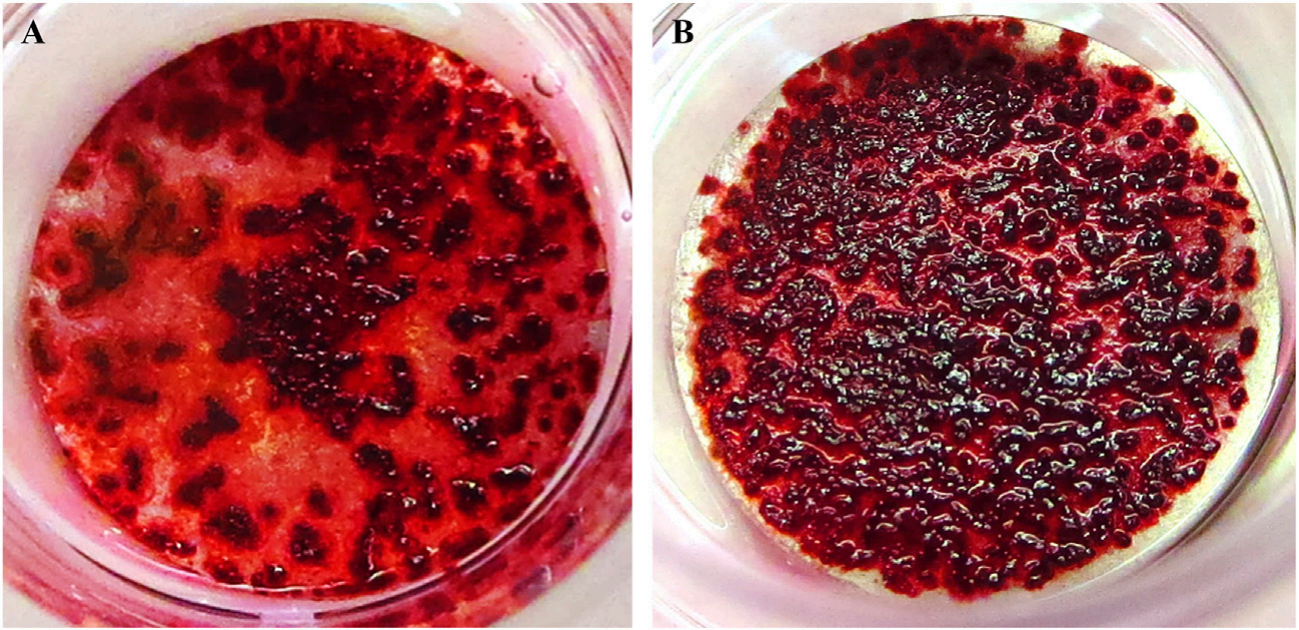
Calcification induction test on the Ti disk surface. The cells were Saos-2. **(A)** Uncoated Ti disk (control). **(B)** Ti disk coated with 1% phosphorylated pullulan.

Compared with the uncoated Ti disks, dense calcification occurred on the surface of the phosphorylated pullulan-coated Ti disks (Figure 9).

### 3.5 Effects of phosphorylated pullulan carrying osteogenic factors on Ti disks

**FIGURE 10:**
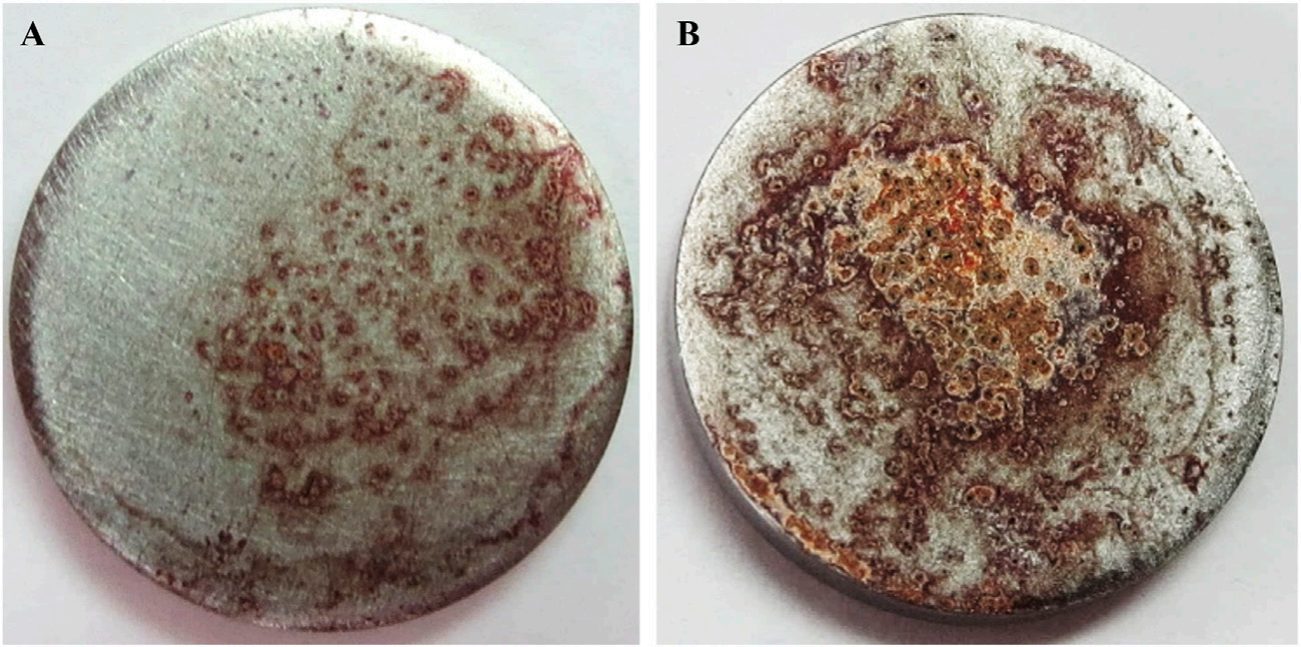
Effects of phosphorylated pullulan carrying osteogenic factor on the Ti disk. The cells were Saos-2. **(A)** Ti disk immersed in 0.2 μg/mL BMP-2 solution. **(B)** Ti disk immersed in 0.2 μg/mL BMP-2 + 1% phosphorylated pullulan solution.

Compared with Ti disks immersed in 0.2 μg/mL BMP-2 solution, the surface of Ti disks immersed in 1% phosphorylated pullulan solution with 0.2 μg/mL BMP-2 showed more calcification and extensive deposition (Figure 10).

### 3.6 Effects on osteoclast-like cell formation

The control group (without phosphorylated pullulan) showed a large number of TRAP-positive cells with three or more nuclei, which has a large round morphology. As the concentration of phosphorylated pullulan in the culture medium increased, the number of nuclei decreased, and the multinucleated giant cells showed a thin and small morphology.

**FIGURE 11:**
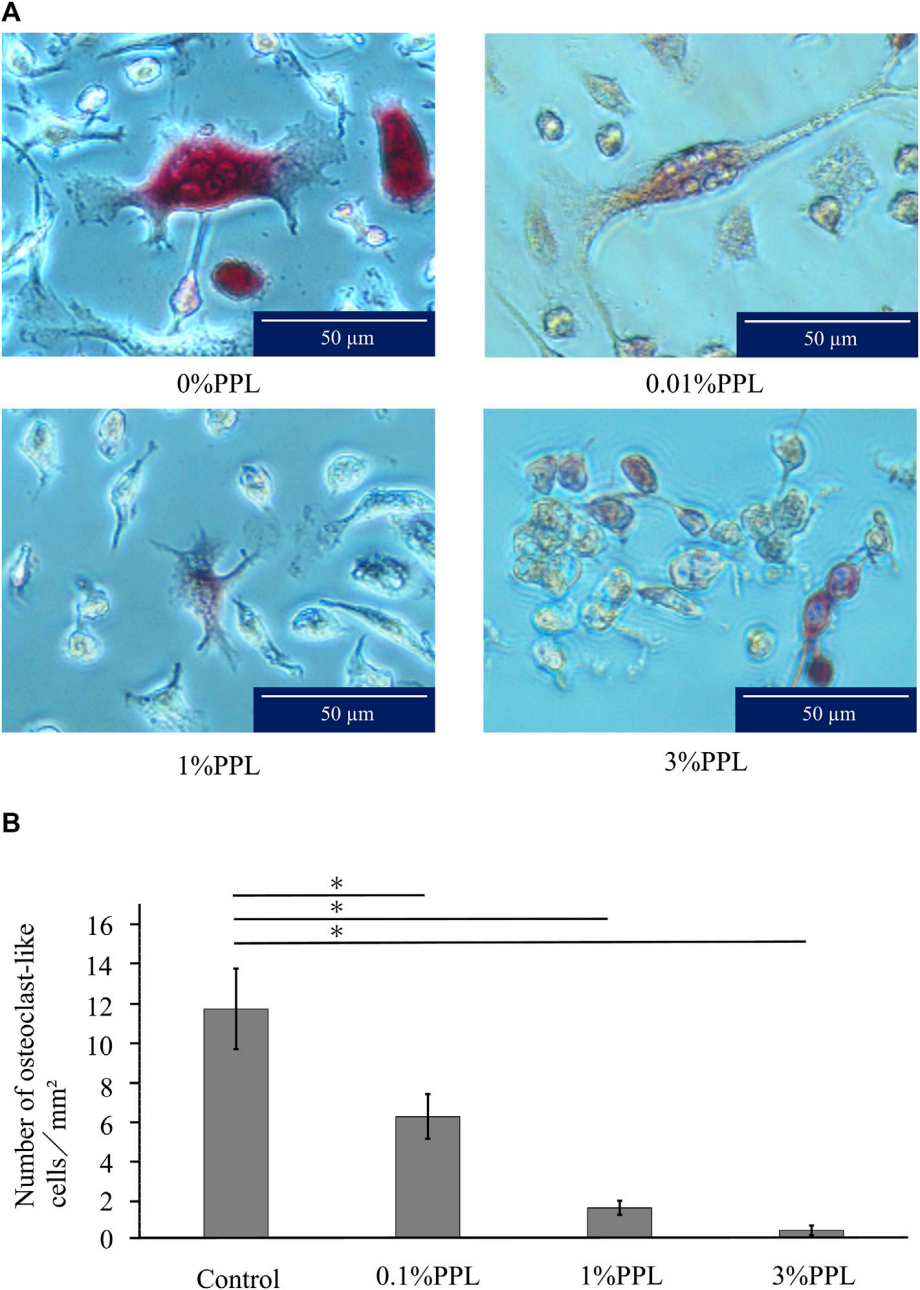
Effect of phosphorylated pullulan on osteoclast-like cell formation. The cells were RAW264.7. **(A)** Observation with an optical microscope. The percentage of phosphorylated pullulan in the culture medium is indicated. **(B)** The measurement of osteoclast-like cells. Significant difference test: *p < 0.05.

The number of osteoclast-like cells was significantly decreased in a concentration-dependent manner in the medium containing 0.1%, 1%, and 3% phosphorylated pullulan compared with the control group (Figure 11).

## 4 Discussion

When culturing cells on a Ti disk surface, examining the surface texture is necessary because the roughness of the Ti disk surface affects cell dynamics (Zizzari et al., 2015; Saruta et al., 2019; Hasegawa et al., 2020). In this study, # 400 waterproof abrasive paper was used as an intermediate roughness with no significant effect on cell adhesion, proliferation, or differentiation. Titanium has also been reported to be a difficult metal to grind (Shimakura et al., 2000). Therefore, in grinding Ti disks, we searched and selected a method that does not cause large scratches on the surface of the disk when the disk is observed by SEM and that allows uniform grinding of the entire surface.

Furthermore, the results of XPS measurements (Figure 4) indicated that once the phosphorylated pullulan was adsorbed to the titanium surface, it adsorbed firmly to the titanium surface without desorbing even under a strong physical cleaning force by ultrasound. In addition to XPS, FT-IR measurement, 31PNMR measurement, and the phosphorus–molybdenum method can be used to detect the presence of phosphorus elements or phosphate groups, but the detection sensitivity of these methods is weak on the topmost surface. XPS is an effective method of analyzing phosphorylated pullulan that is adsorbed to the Ti surface. Titanium combines with oxygen in the atmosphere and easily changes to titanium oxide to form a passivation film (Hanawa, 2022). Consequently, titanium becomes resistant to corrosion, making it an excellent implant material. Based on previous reports, titanium oxide hydrate and phosphate ions are adsorbed and affected by pH (Healyt and Ducheyne, 1992; Kang et al., 2011). Another report indicates that the phosphate group formed a strong chemical bond with titanium oxide, such as a chelate bond, following pH (Iravani et al., 2015). Thus, it is predicted an electrical or some other type of attraction occurred between titanium oxide and the phosphate group of phosphorylated pullulan. In addition, a detailed investigation of pH and other conditions may enable thicker phosphorylated pullulan films to adsorb Ti disk surfaces with a strong chemical bond. Further analysis of the adhesion mechanism between titanium dioxide and phosphate groups is required in the future.

In the cell proliferation test, the addition of phosphorylated pullulan to the culture medium slightly decreased cell proliferation. On the one hand, when phosphorylated pullulan was coated onto the Ti disk surface, the number of cells increased compared with the control. This trend may be due to the phosphorylated pullulan adsorption of molecules and ions. Phosphorylated pullulan on the Ti disk surface chemically or physically could adsorb carbohydrates, lipids, amino acids, vitamins, and various factors that act on cells in a culture medium (Warner, 2019). Thus, phosphorylated pullulan adsorbing to the Ti disk surface continuously may supply high-concentration functional molecules to the cells adhering to the Ti disk surface. On the other hand, when phosphorylated pullulan was added directly to the culture medium, the supply of molecules that act on the cells could be reduced for the cells adhering to the bottom of the culture dish. Molecules that act on cells could be adsorbed by phosphorylated pullulan, which is suspended in the culture medium. Consequently, cell proliferation decreased gradually as the concentration of phosphorylated pullulan increased.

A similar mechanism is suggested for the calcification induction test and the formation of osteoclast-like cells. The phosphorylated pullulan in the culture medium might adsorb dexamethasone, ascorbic acid, β-glycerophosphate, which are necessary for calcification, and RANKL, which is necessary for osteoclast-like cell formation. Therefore, these substances adsorbed by the phosphorylated pullulan in the culture medium could not reach the cells attached to the bottom of the culture dish. In addition, calcification induction and the formation of osteoclast-like cells did not occur sufficiently. This phenomenon may result in the suppression of calcification and a decrease in the number of osteoclast-like cells. On the contrary, in the case of phosphorylated pullulan on the Ti disk, the continuous and high-concentration supply of dexamethasone, ascorbic acid, and β-glycerophosphate adsorbed by phosphorylated pullulan to cells could promote calcification. However, it is unlikely that the phosphorylated pullulan-treated Ti disk was stained because of the adsorption of alizarin red to the phosphorylated pullulan. Considering that the structure of alizarin red consists of three benzene rings and two functional hydroxyl groups, the hydroxyl groups could be negatively charged in an aqueous solution. Therefore, it has the same charge as the phosphorylated pullulan. Thus, they could repel each other.

Consequently, cells can directly and continually receive molecules that act on cells in the culture medium and substances necessary for calcification from phosphorylated pullulan by selectively adhering phosphorylated pullulan to a target site such as a Ti disk.

The three elements involved in bone regeneration are scaffolding, cells, and growth factors. (Pilipchuk, 2015; Thrivikraman et al., 2017). In addition to surface modification of implants, which is related to scaffolding, this study also investigated growth factors. Furthermore, the use of growth factors can be an approach to a good improvement method for successful implant treatment. One bone growth factor is BMP-2. However, BMP-2 alone is not easily maintained on the Ti disk surface; therefore, a carrier with biodegradability, sustained release, and easy moldability is necessary when BMP-2 is applied to a living body (Bialy et al., 2017; Kowalczewski and Saul, 2018). Thus, we focused on the adsorb property of pullulan itself and the phosphate groups of phosphorylated pullulan. BMP-2 has a positive charge under a neutral solution (Guzman et al., 2015). If phosphate groups of phosphorylated pullulan ionize, it might electrically attract BMP-2. We thought that BMP-2 could be chemically or physically loaded on phosphorylated pullulan using these two properties. In fact, calcification was enhanced on Ti disks treated with a mixed solution of phosphorylated pullulan and BMP-2 compared with Ti disks treated only with BMP-2.

When phosphorylated pullulan bound to BMP-2 adsorbed to titanium, Saos-2 on the disk surface directly, continuously, and at high concentrations could receive BMP-2, dexamethasone, ascorbic acid, and β-glycerophosphate. Therefore, the formation of calcification on the Ti disks could be promoted. These results indicate that BMP-2 could be loaded onto phosphorylated pullulan without losing its function. However, excessive amounts of BMP-2 have a negative effect on calcification (Wikesjö et al., 2008; Chaudhar et al., 2012). Thus, the amount of BMP-2 used should be noted.

In current hydroxyapatite coatings, hydroxyapatite is not absorbed in the tissue when it is detached from the surface, so it remains for a long time and it can easily become a source of infection. However, phosphorylated pullulan treatment could chemically bond to titanium, and it has high adhesion compared with hydroxyapatite coating. In addition, phosphorylated pullulan treatment does not become a source of infection because phosphorylated pullulan is gradually replaced by bone when implanted in bone (Morimoto et al., 2023). It is relatively quickly absorbed, although it is detached. Infection is harmful to implant treatment, and phosphorylated pullulan treatment is considered to be a useful treatment method.

## 5 Conclusion

Based on the properties of phosphorylated pullulan, the effects of phosphorylated pullulan on cells and its function as a drug carrier in cell culture were examined.

The results showed that phosphorylated pullulan, when adsorbed to Ti disks, promotes the proliferation of osteoblast-like cells and induces calcification on the disks.

The drug carrier function of phosphorylated pullulan with BMP-2 was also shown to induce calcification on Ti disks.

The addition of phosphorylated pullulan to the culture medium affected the formation of osteoclast-like cells.

Therefore, phosphorylated pullulan affects the proliferation and differentiation of bone-associated cells depending on the application conditions. Moreover, the coating of phosphorylated pullulan on titanium implants may contribute to the improvement of osseointegration and the development of surface treatment methods for implants.

## Data Availability

The original contributions presented in the study are included in the article/Supplementary Material, further inquiries can be directed to the corresponding author.
